# Abstracts from Foreign Journals

**Published:** 1903-03

**Authors:** S. J. J. Harger

**Affiliations:** Philadelphia, Pa.


### ABSTRACTS FROM FOREIGN JOURNALS.

### FRENCH.

Under the Direction of S. J. J. Harger, V.M.D.,
PHILADELPHIA, PA.

Veterinary Students in Italy. During the school year of 1901–02 over 1200 students attended the veterinary schools of Italy. These were distributed as follows: Naples, 269; Turin, 212; Milan, 200; Bologne, 199; Pisa, 109; Perouse, 74; Modena, 53; Parme, 45; Camerino, 42. The opinion prevails that there are more schools in Italy than the needs of the country demand.—*Rec. de Med. Vet.*

Hæmopneumothorax from Injury to the Ribs. Darrow reports a horse that was kicked by another horse over the ribs. The consecutive symptoms were a hot, painful swelling, dyspnœa, temperature, 39°. Within the wound was found an enormous blood-clot, fracture of the eighth rib, and perforation of the pleura. Three days afterward the inferior third of the pleural sac was filled with exudation; above there was increased resonance on account of air having entered the pleural cavity. Thoracentesis was practised at intervals, and an abundant aseptic liquid was removed, which became less at every operation. Finally the increased resonance disappeared and the recovery was complete, due essentially to the non-infection of the pleura after the injury.—*Rev. Vet.*

Bovine Tuberculosis from Ingestion of Tubercular Material. Schottelius, of the Institute of Hygiene of Freiburg, experimented upon the transmission of human tuberculosis to cattle by feeding them upon expectorated material. The experiment involved a cow, and two calves six months old. One cow and a calf were reserved as checks. Each animal received during three months tubercular matter twenty-four times, obtained from persons in advanced stages of tuberculosis. This tubercular material, in quantity of 50 grammes, was mixed with the feed. During the course of the experiment nothing abnormal was observed. The necropsy of the cow showed a tubercular enteritis, with considerable tumefaction of the mesenteric lymphatic glands and caseation of the mediastinal and bronchial glands; there were also found caseous pneumonia of a tubercular nature and isolated miliary tubercles on the pleura. In the calves the submaxillary ganglia were much tumefied and caseous, and some of the mesenteric glands were tubercular.

Microscopic examination showed the presence of the tubercle bacillus in the diseased organs of these three animals. The organs of the check animals were normal.

From these facts Schottelius concludes that human tuberculosis is transmissible to the bovine species, and that this disease in man is identical with that in cattle.—*Munch. med. Wochensch.*

Resection of Synovial Membranes in the Horse. The operation is applicable to dilatations of the synovial sheaths of tendons (windgalls). The technique of the operation is as follows: (1) Excision of a fusiform flap of skin; (2) dissection of the synovial membrane; (3) excision of a flap of synovial membrane; (4) flushing of the synovial sac with a hot solution of phenic acid; (5) suturing of the synovial membrane with catgut; (6) suturing of the skin; (7) dressing of gauze and sterilized cotton.

*Observation.*—Draught-mare with a large windgall on the posterior right pastern, causing lameness.

Operated September 20, 1899. Excision of a flap of skin, 7 cm. long and 2.5 cm. wide, a trifle behind the axis of the tumor, to avoid the digital vessels and nerves; dissection of the subcutaneous connective tissue; puncture of the synovial sac with the bistoury, from which escaped grumous synovial fluid, and removal with the scissors of the accessible part of the membrane; fixation of the borders with artery forceps and flushing of the synovial sac; suture of the synovial membrane with catgut, and of the skin with Florence hair; dressing. September 21, no fever; no weight on foot. September 22, foot bearing some weight. September 23, foot bearing considerable weight. Not any change supervened until October 10, when the dressing was removed. The tumor had disappeared and the incision cicatrized. The sutures were removed and the parts redressed. Dressing removed October 30 and the mare sent to work. The windgall did not return.

Meynard and Moreau operated upon five cases. In three the result was permanent; in one the swelling returned; in the fifth the immediate cure was complete, but the animal had to be destroyed on account of paralysis from causes not stated, although prior to this accident the animal was able freely to bear weight on the foot.

The most vigorous antisepsis must be observed. Calvé demonstrated that this can be done by operating on a litter covered with a rubber mattress. Maud M. fostered the animal in stocks (Vinsal). —*Rev. Gen. de Med. Vet.*

